# Spontaneous incorporation of gold in palladium-based ternary nanoparticles makes durable electrocatalysts for oxygen reduction reaction

**DOI:** 10.1038/ncomms11941

**Published:** 2016-06-23

**Authors:** Deli Wang, Sufen Liu, Jie Wang, Ruoqian Lin, Masahiro Kawasaki, Eric Rus, Katharine E. Silberstein, Michael A. Lowe, Feng Lin, Dennis Nordlund, Hongfang Liu, David A. Muller, Huolin L. Xin, Héctor D. Abruña

**Affiliations:** 1 Key laboratory of Material Chemistry for Energy Conversion and Storage (Huazhong University of Science and Technology), Ministry of Education, Hubei Key Laboratory of Material Chemistry and Service Failure, School of Chemistry and Chemical Engineering, Huazhong University of Science and Technology, Wuhan 430074, China; 2 Center for Functional Nanomaterials, Brookhaven National Laboratory, Upton, New York 11973, USA; 3 JEOL USA, Inc., Peabody, Massachusetts 01960, USA; 4 Department of Chemistry and Chemical Biology, Cornell University, Ithaca, New York 14853, USA; 5 Energy Storage and Distributed Resources Division, Lawrence Berkeley National Laboratory, Berkeley, California 94720, USA; 6 Stanford Synchrotron Radiation Lightsource, SLAC National Accelerator Laboratory, Menlo Park, California 94025, USA; 7 School of Applied and Engineering Physics, Cornell University, Ithaca, New York 14853, USA; 8 Kavli Institute at Cornell for Nanoscale Science, Cornell University, Ithaca, New York 14853, USA

## Abstract

Replacing platinum by a less precious metal such as palladium, is highly desirable for lowering the cost of fuel-cell electrocatalysts. However, the instability of palladium in the harsh environment of fuel-cell cathodes renders its commercial future bleak. Here we show that by incorporating trace amounts of gold in palladium-based ternary (Pd_6_CoCu) nanocatalysts, the durability of the catalysts improves markedly. Using aberration-corrected analytical transmission electron microscopy in conjunction with synchrotron X-ray absorption spectroscopy, we show that gold not only galvanically replaces cobalt and copper on the surface, but also penetrates through the Pd–Co–Cu lattice and distributes uniformly within the particles. The uniform incorporation of Au provides a stability boost to the entire host particle, from the surface to the interior. The spontaneous replacement method we have developed is scalable and commercially viable. This work may provide new insight for the large-scale production of non-platinum electrocatalysts for fuel-cell applications.

## Abstract (web summary)

Replacement of platinum is important for lowering the cost of fuel-cell electrocatalysts, but less precious alternatives such as palladium are hindered by lower durability. Here, the authors show that incorporation of trace amounts of gold improves the durability of palladium based oxygen reduction catalysts.

## Graphical abstract

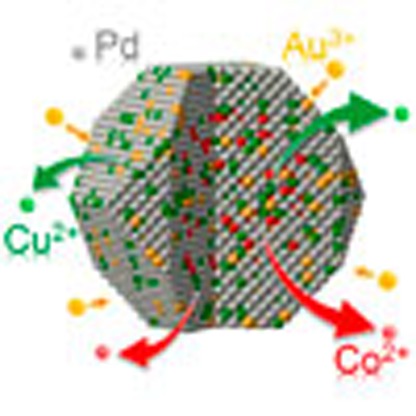

Pt-based nanoparticles have been extensively studied as electrocatalysts for the oxygen reduction reaction (ORR) in both acid and alkaline media^1^,^2^,^3^,^4^,^5^,^6^,^7^. However, the high cost and the limited reserve of Pt hinder the widespread deployment of fuel-cell technologies. Numerous strategies have been employed including developing new structures and morphologies for Pt-based nanocatalysts^3^,^8^,^9^,^10^,^11^,^12^,^13^, Pt monolayer decorated nanoparticles^14^,^15^, other platinum group nanomaterials^16^,^17^,^18^,^19^, as well as non-precious-metal nanoparticles^16^,^20^,^21^,^22^,^23^,^24^. Among these alternatives, Pd is very attractive because it is much more abundant and less expensive than Pt. However, the ORR activity of pure Pd in acid is much lower than that of Pt. Previous studies have focused on alloying Pd with 3*d* transition metals, such as Fe^25^, Co^14^, Ni^26^, Cu^27^ and others, resulting in a significant improvement in catalytic activity. Moreover, the activity of Pd-based binary nanoparticles can be further enhanced by adding a third element^28^. In spite of that, Pd-based catalysts undergo fast degradation as ORR cathodes under regular cycling conditions. Previous studies have shown that these catalysts can be stablized by galvanically replacing the surface atoms with Pt^14^,^29^, which simultaneously increases Pt usage.

In this study, we show that Au atoms can penetrate the bulk lattice of Pd_6_CoCu nanoparticles and spontaneously replace Co and Cu atoms in the interior of the nanoparticles through galvanic replacement reactions. This effect allows the uniform incorporation of trace amounts of Au in Pd_6_CoCu nanoparticles, which enhances the long-term stability of the electrocatalyst for the ORR. We also show that there a is much smaller displacement (ca. 25 mV) in the half-wave potential for Au–Pd_6_CoCu nanoparticles over 10,000 voltage cycles in O_2_-saturated 0.1 M HClO_4_ solution, compared with a 54-mV negative potential shift for Pd_6_CoCu nanoparticles without Au incorporation after only 1,000 cycles in O_2_-saturated 0.1 M HClO_4_ solution. More importantly, PEMFC single-cell performance using aAu–Pd_6_CoCu/C cathode electrocatalyst exhibits a maximum power-density loss of about 21% after a 100-h stress test at 80 °C, which is dramatically better than a Au-free catalyst.

## Results

### Synthesis and charaterization of Pd_6_CoCu/C nanoparticles

**Figure 1:**
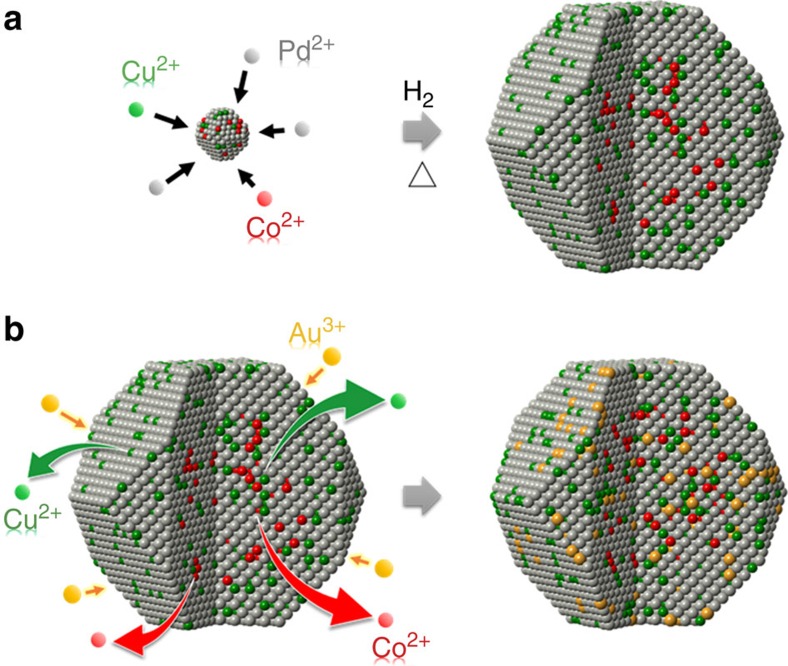
Diagrammatic illustration of the synthetic strategy of nanoparticles. Schematic illustration of the formation of Pd_6_CoCu/C (**a**) and Au–Pd_6_CoCu/C (**b**).

Pd_6_CoCu nanoparticles supported on Vulcan XC-72 carbon (Pd_6_CoCu/C) were synthesized using an impregnation reduction method followed by high temperature heat-treatment as reported previously^8^,^14^,^30^. The crystal structures of the particles were characterized by X-ray diffraction (Supplementary Fig. 1). Pd/C, Pd_3_Cu/C and Pt_3_Co/C nanoparticles are also shown for comparison. The X-ray diffraction patterns suggest a face-centered cubic structure. The peak positions of the bi-/tri- metallic nanoparticles shift to higher angles, as anticipated, compared to pure Pd/C. The angular shifts indicate alloy formation between Pd and Co/Cu, and reflect a lattice contraction, which is caused by the incorporation of smaller atoms, Co/Cu, into the Pd face-centered cubic lattice. The surface composition of the particles was determined by X-ray photoelectron spectroscopy (XPS) (Supplementary Fig. 2). For the Pd_6_CoCu/C sample, there is almost no Co2*p* signal, while two distinct Cu2*p* peaks are evident. This suggests that Cu is likely distributed closer to the surface of the particle than Co as illustrated in Fig. 1.

**Figure 2:**
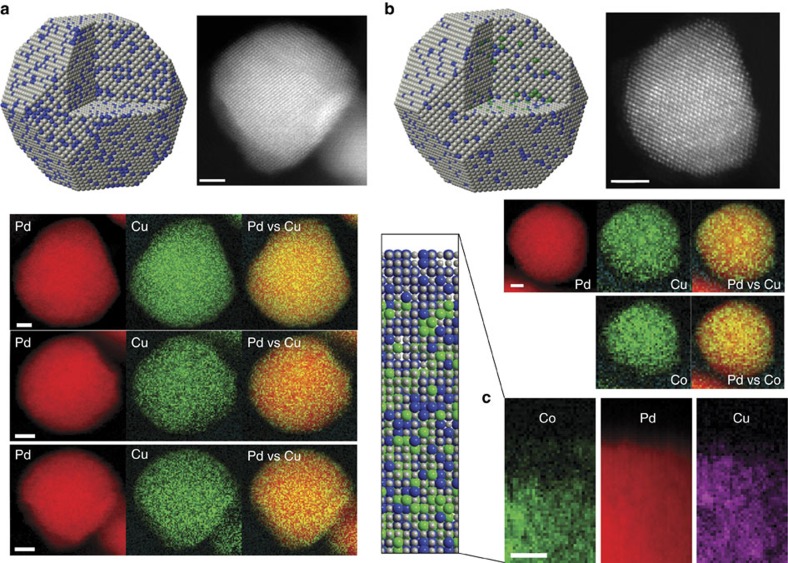
ADF–STEM images and elemental mapping. Modelled structure, ADF-STEM image, and 2D EELS maps of each element and composites of Pd3Cu/C (**a**) and Pd6CoCu/C (**b**). Scale bar, 2 nm. (**c**) Elemental maps of a Pd_6_CoCu particle's surface and subsurface volume extracted from an atomic-scale EELS map. Scale bar, 1 nm.

To directly measure the structure and chemical composition of the nanoparticles at the atomic scale, we used aberration-corrected scanning transmission electron microscopy (STEM) in conjunction with electron energy loss spectroscopy (EELS) to characterize the samples (Fig. 2). The Pd_6_CoCu/C nanoparticles appear to be uniformly distributed on the carbon support with an average particle size of 7.5 nm, consistent with the particle size calculated from X-ray diffraction (Supplementary Fig. 3 and Supplementary Table 1). Figure 2a,b show the annular dark-field (ADF)-STEM images, which reflect the atomic mass contrast of the materials, and EELS elemental mapping of the as-prepared Pd_3_Cu/C and Pd_6_CoCu/C nanoparticles, respectively. For Pd_3_Cu/C nanoparticles, the EELS spectroscopic images in Fig. 2a indicate a uniform elemental distribution of Pd and Cu from the particle's interior to the surface. There is no surface segregation between Pd and Cu. This is in contrast to our previously reported Pd_3_Co/C nanoparticles that had a ∼1-nm-thick Pd-rich shell on the particles surface^14^,^30^.

Figure 2b shows the ADF-STEM images and EELS elemental maps of Pd_6_CoCu/C nanoparticles. There is no phase segregation in the composite maps of Pd and Cu, which is the same as in Pd_3_Cu/C nanoparticles. However, a Pd-rich shell was observed for the composite maps of Pd and Co. To quantify the surface and subsurface chemical composition with atomic-scale resolution, we investigated a single facet of a Pd_6_CoCu particle (Fig. 2c). The chemical maps in Fig. 2c clearly show that there is a ∼1-nm-thick Pd–Cu-rich layer present on the surface of the Pd_6_CoCu/C nanoparticle. This is consistent with the low Co 2*p* signal in XPS, as the Pd–Cu-rich shell blocks the photoelectrons generated from Co 2*p* core levels.

The difference in the segregation behaviour between Pd–Cu and Pd–Co is likely a result of two contributions. First, as has been calculated by Nørskov *et al*.^31^ and Wang *et al*.^32^, the surface segregation energy between Pd and Co is much higher than between Pd and Cu. This suggests that there would be a much stronger driving force for surface segregation in Pd–Co than in Pd–Cu nanoparticles. Second, the differences in the gas-adsorption energies of two metals can induce surface segregation^33^. The particles were heat-treated in a flowing stream of hydrogen. The fact that hydrogen binds strongly to Pd surfaces and can even be stored in Pd lattices, suggests that Pd can be enriched at the surface, thus pushing other elements away from the surface.

### Synthesis and charaterization of Au–Pd_6_CoCu/C nanoparticles

The Au cluster-modified Pt/C nanoparticles, prepared by the Cu under-potential deposition method, have been reported to significantly enhance the stability of Pt/C for the ORR^34^. Recently, Stamenkovic *et al*. reported that Au-stabilized Pt–Au–Ni nanoparticles with a multilayer profile of Ni@Au@PtNi could endure extended durability testing^35^. Adzic *et al*. showed that structurally ordered intermetallic AuPdCo nanoparticles exhibited increased activity and much better durability for the ORR^36^.

Inspired by previous studies, we used a galvanic replacement method to deposit Au on Pd_6_CoCu/C nanoparticles since the equilibrium electrode potential of the AuCl_4_^−^/Au couple (0.93 V versus standard hydrogen electrode (SHE)) is more positive than those of the Cu^2+^/Cu (0.34 V versus SHE) and PdCl_4_^2−^/Pd (0.591 V versus SHE) couples. The stability of Pd_6_CoCu/C nanoparticles for ORR could be enhanced after Au replacement. The deposition procedure is similar to the one that we have previously reported^14^,^30^. XPS spectra clearly show Au_4f_ peaks (inset in Supplementary Fig. 4), indicating that Au has successfully replaced atoms on/in the Pd_6_CoCu/C nanoparticle. Moreover, there is no evidence of a phase transformation after Au has been deposited as shown by X-ray diffraction measurements (Supplementary Fig. 5).

**Figure 3:**
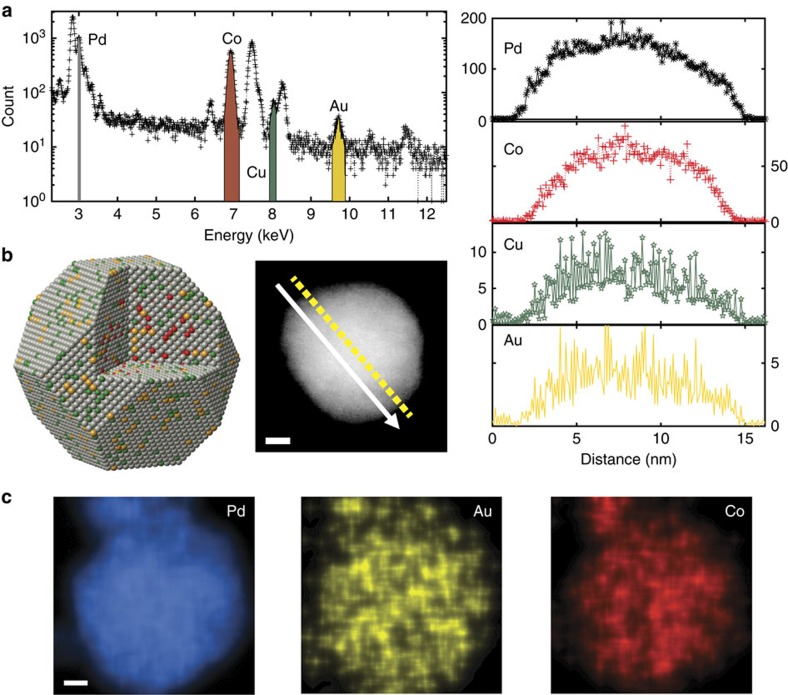
Composition analysis of a Au–Pd_6_CoCu/C nanoparticle. (**a**) Energy-dispersive X-ray spectroscopic (EDX) analysis of Au–Pd_6_CoCu/C nanoparticles. (**b**) Elemental distribution of Pd, Co, Cu and Au in a single Au–Pd_6_CoCu/C nanoparticle extracted from an aberration-corrected STEM–EDX line profile. Scale bar, 2 nm. (**c**), Aberration-corrected STEM–EDX 2D elemental maps of Pd, Co and Au. Scale bar, 5 nm.

Figure 3a shows energy-dispersive X-ray spectroscopic analysis of Au–Pd_6_CoCu/C nanoparticles in an aberration-corrected STEM. A line-profile was acquired on a single particle to reveal the elemental distribution of Pd, Co, Cu and Au using a 200 keV aberration-corrected STEM (Fig. 3b). Interestingly and surprisingly, Au showed the same distribution profile as Pd, suggesting that Au penetrates through the entire particle as illustrated in Fig. 1. To demonstrate the elemental distribution of Au without ambiguity, we performed a two-dimensional (2D) mapping of the particle. The Au map shows the same spatial distribution as Pd, again strongly supporting our assertion that Au atoms are uniformly incorporated into the nanoparticle (Fig. 3c).

**Figure 4:**
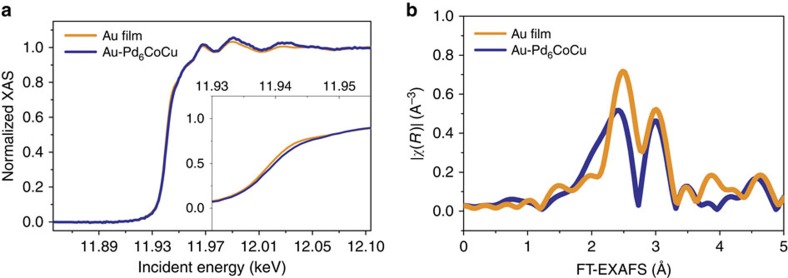
X-ray absorption analysis. (**a**) XANES and (**b**) FT-EXAFS spectra of Au-L3 edge in a Au film and a Au–Pd_6_CoCu/C nanoparticle. EXAFS, extended X-ray absorption fine structure; XANES, X-ray absorption near-edge structure.

The incorporation of Au was further examined by X-ray absorption spectroscopy (XAS). The Au-L3 X-ray absorption near-edge structure was used to discriminate between highly oxidized and reduced forms of the metal. Figure 4a compares a Au film and Au–Pd_6_CoCu/C nanoparticles. It can be clearly seen that both samples exhibit the characteristic three-peak pattern following the edge jump^37^, indicating that the gold in the Au–PdCuCo sample is reduced (metallic). The ‘whiteline' intensity of Au, which is closely related to the d-band population, was slightly lower in Au–Pd_6_CoCu/C than that in the Au film, and the edge position was shifted to slightly higher energy. Most importantly, the small but significant decrease in intensity of the whiteline demonstrates a partial charge transfer from Pd to Au as observed and described in detail for Au–Pd alloys in previous studies^38^,^39^,^40^.

The Fourier transform of the extended X-ray absorption fine structure of the Au film and Au–Pd_6_CoCu/C nanoparticles, measured at the Au-L3 edge, are shown in Fig. 4b. The two peaks in the spectra indicate that gold has two different near neighbours. We ascribe the decrease in magnitude of the first-shell peak in Au–Pd_6_CoCu/C relative to the gold foil to a lower coordination number of Au in the nanoparticle, which is consistent with a previous XAS study of Pd–Au alloys by Sham^38^ and a consequence, at least in part, of the phase shifts of high-Z absorbers and scatterers. In a word, the XAS study demonstrates that at the bulk ensemble level, Au has been uniformly incorporated in the Pd–Co–Cu lattice, rather than forming a Au shell or Au clusters.

The observation that Au can spontaneously penetrate the Pd–Co–Cu lattice to reach the interior of the particle might be related to the fact that the as-prepared Pd_6_CoCu/C nanoparticles were Pd and Cu rich on the surface (Fig. 2c). The equilibrium electrode potential of the Cu^2+^/Cu couple is lower than that of the PdCl_4_^2−^/Pd couple. Therefore, it is likely that Au displaces Cu first, leaving the Pd atoms untouched. In addition, Au and Pd are intermixable at all proportions. Therefore, thermodynamically, it is favourable for Au to uniformly mix with Pd. This is in contrast to the previously investigated Pt–Pd_3_Co system^14^,^30^, where Pt can only displace atoms on the surface of the nanoparticles and form a Pt-rich shell. The phase diagram shows that, in this case, Pt and Pd are not miscible in all proportions. With regards to the Pt-displacement experiment, we speculate that the intersolubility of Au and Pd might be one of the driving forces for Au to penetrate the Pd–Co–Cu bulk lattice and thus the entire nanoparticle.

### Electrochemical and fuel-cell testing

**Figure 5:**
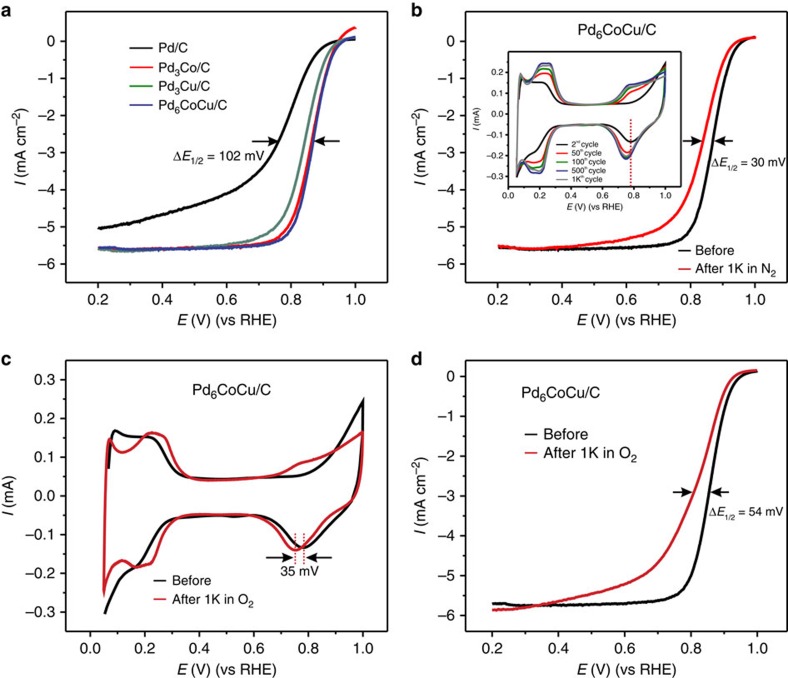
Electrocatalytic activity for ORR. (**a**) Comparison of ORR polarization curves for Pd/C, Pd_3_Co/C, Pd_3_Cu/C and Pd_6_CoCu/C in O_2_-saturated 0.1 M HClO_4_ at room temperature; rotation rate, 1,600 r.p.m.; and sweep rate, 5 mV s^−1^. (**b**) ORR polarization curves for Pd_6_CoCu/C before and after 1,000 potential cycles in N_2_-saturated 0.1 M HClO_4_, rotation rate, 1,600 r.p.m. and sweep rate, 5 mV s^−1^. The inset in **b** shows the changes in the CV profiles after different numbers of cycles at a sweep rate of 50 mV s^−1^. (**c**) CV changes in the voltammetric profile for Pd_6_CoCu/C before and after 1,000 potential cycles in O_2_-saturated 0.1 M HClO_4_ between 0.6 and 1.0 V at a sweep rate of 50 mV s^−1^. (**d**) ORR polarization curves for Pd_6_CoCu/C before and after 1,000 potential cycles in O_2_-saturated 0.1 M HClO_4_, rotation rate, 1,600 r.p.m.; sweep rate, 5 mV s^−1^.

The ORR activities of the electrocatalysts were evaluated in an O_2_-saturated 0.1 M HClO_4_ solution by loading the materials (with the same Pd mass loading) onto a glassy carbon rotating disk electrode with experiments performed at a rotation rate of 1,600 r.p.m. and a sweep rate of 5 mV s^−1^ at room temperature. All electrodes were pretreated by cycling the potential between +0.05 and +1.00 V at a sweep rate of 50 mV s^−1^, for 50 cycles, before the ORR activity test. Polarization curves for the ORR of the different catalysts are shown in Fig. 5a. The ORR kinetics was markedly accelerated on the tri-metallic Pd_6_CoCu/C alloy nanoparticles, and the half-wave potential (*E*_1/2_) was significantly positively shifted, relative to Pd/C and Pd_3_Cu/C nanoparticles, indicating a significant increase in the ORR activity. It can also be seen in Fig. 5a that the Pd_6_CoCu/C nanoparticles exhibit a slightly higher ORR activity than the Pd_3_Co/C nanoparticles. This may be due to the smaller particle size and slightly different lattice parameter (Supplementary Table 1).

The durability of the Pd_6_CoCu/C nanoparticles was evaluated by potential cycling between +0.05 and +1.0 V at a scan rate of 50 mV s^−1^ for 1,000 cycles in N_2_-saturated 0.1 M HClO_4_ solution followed by an assessment of the ORR activity. As shown in Fig. 5b, after 1,000 potential cycles, the Pd_6_CoCu/C catalyst showed a degradation of around 30 mV in its half-wave potential for the ORR. The CVs after different numbers of cycles are presented in the inset to Fig. 5b. Durability tests were also conducted in O_2_-saturated 0.1 M HClO_4_ solution between +0.6 and +1.0 V at a scan rate of 50 mV s^−1^ for 1,000 potential cycles. It can be seen from changes in the CVs (Fig. 5c) that the hydrogen adsorption/desorption peaks shifted to positive potentials, while the peak potential for the reduction of the Pd oxides shifted negatively by ∼35 mV. The ORR durability, presented in Fig. 5d, shows a significant negative shift in the half-wave potential of about 54 mV after 1,000 potential cycles, indicating that the decay in the catalytic activity of Pd_6_CoCu/C was accelerated when cycling in the presence of oxygen.

The decay in electrocatalytic activity for the ORR on Pd_6_CoCu/C electrode, after continuous cycling, most likely arises from surface changes during cycling. Since the initial surface of the Pd_6_CoCu/C nanoparticles is a Pd–Cu-rich alloy (Fig. 2b,c; Pd and Cu are evenly distributed on the surface), the Cu dissolves during potential cycling, causing the lattice strained Pd surface to expand with Cu leaching out of the nanoparticle surface. The surface composition changes can be seen clearly from the CVs of the Pd_6_CoCu/C nanoparticle after different cycles (inset of Fig. 5b,c). The onset of Pd oxide formation, and the Pd oxides reduction peaks shift towards negative potentials with cycling. The readily formed Pd oxides inhibit the OH_ad_ species on the Pd_6_CoCu/C electrode surface and block the active sites for O_2_ adsorption, causing the decay in the ORR activity.

**Figure 6:**
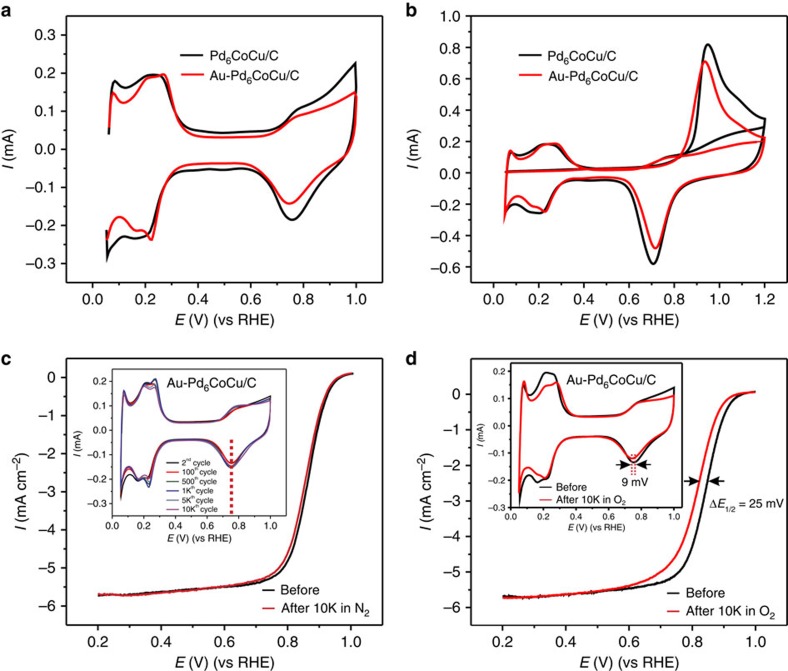
Electrochemical measurements. (**a**) CVs and (**b**) CO stripping of Pd_6_CoCu/C nanoparticles with and without Au decoration in 0.1 M HClO_4_ purged with N_2_, sweep rate, 50 mV s^−1^. (**c**) ORR polarization curves of Au–Pd_6_CoCu/C nanoparticles before and after cycling in N_2_-saturated 0.1 M HClO_4_; rotation rate, 1,600 r.p.m.; and sweep rate, 5 mV s^−1^. The inset in **c** shows CVs of the Au–Pd_6_CoCu/C nanoparticles after different numbers of potential cycles between 0.05 and 1.0 V in N_2_-saturated 0.1 M HClO_4_ and sweep rate, 50 mV s^−1^. (**d**) ORR polarization curves of Au–Pd_6_CoCu/C nanoparticles before and after cycling in O_2_-saturated 0.1 M HClO_4_ between 0.6 and 1.0 V; rotation rate, 1,600 r.p.m. and sweep rate, 5 mV s^−1^. The inset in **d** shows changes in the voltammetric profile for Au–Pd_6_CoCu/C before and after 10,000 potential cycles between 0.6 and 1.0 V in O_2_-saturated 0.1 M HClO_4_, sweep rate, 50 mV s^−1^.

After replacement with Au the CV of the Pd_6_CoCu/C nanoparticle changed significantly. As shown in Fig. 6a, the total surface area decreased (see also the CO stripping in Fig. 6b) and the double layer current also decreased. In the hydrogen region, the Au-decorated Pd_6_CoCu nanoparticles exhibited, two well-defined hydrogen adsorption/desorption peaks compared with one broad hydrogen peak for Pd_6_CoCu/C nanoparticles, from +0.1 to +0.3 V. In addition, the Pd oxides reduction peak shifted slightly negative after Au decoration. Because the Au atom is larger than Pd, the lattice parameter of Pd on the top layer of the nanoparticle will expand after Au incorporation. The expanded lattice will modify the oxygen adsorption energy of the nanocatalyst, causing the negative shift of the oxides reduction peak. The same phenomenon can also be seen in the CO-stripping voltammetry (Fig. 6b), which shows that the onset and peak potential for CO oxidation, shifted slightly negative after Au-decoration of the Pd_6_CoCu/C nanoparticles.

The stabilizing effect of Au on the Pd_6_CoCu/C nanoparticles was evaluated by cycling the electrode in both N_2_- and O_2_-saturated 0.1 M HClO_4_ solutions, respectively. As shown in the inset of Fig. 6c,d, the CVs of the Au–Pd_6_CoCu/C nanoparticles in an N_2_ atmosphere changed slightly with increasing number of potential cycles. Moreover, unlike the Pd_6_CoCu/C catalysts, the peak position of the Pd oxides reduction remained unchanged in the N_2_ atmosphere, although it shifted negatively by about 9 mV in the O_2_ atmosphere after 10,000 potential cycles, indicating that the Pd_6_CoCu/C catalyst was stabilized by the Au decoration. Besides, the morphology of the Au–Au–Pd_6_CoCu/C nanoparticles remained almost spherical after potential cycles, although the average size was slightly increased (Supplementary Fig. 6). The catalytic activity for the ORR of the Au–Pd_6_CoCu/C catalyst exhibited a slight degradation in the half-wave potential after 10,000 cycles in N_2_-saturated 0.1 M HClO_4_ solution between +0.05 and +1.0 V (Fig. 6c). In contrast, the Pd_6_CoCu/C catalyst exhibited a shift of ∼30 mV (Fig. 5b). After testing the electrode for 10,000 potential cycles in O_2_-saturated 0.1 M HClO_4_ solution between +0.6 and +1.0 V at a scan rate of 50 mV s^−1^ (Fig. 6d), the electrochemical surface area decreased by only 13.8% when compared with the initial value. The half-wave potential shifted negatively by about 25 mV, a value that is much lower than that of the Pd_6_CoCu/C electrode, after only 1,000 cycles (54 mV). The enhancement in the stability likely derives from the partial charge donation of Pd to Au (Fig. 4a) and slight expansion of the Pd lattice (Fig. 6a; Supplementary Table 1), thus enhancing the durability of Pd during cycling.

**Figure 7:**
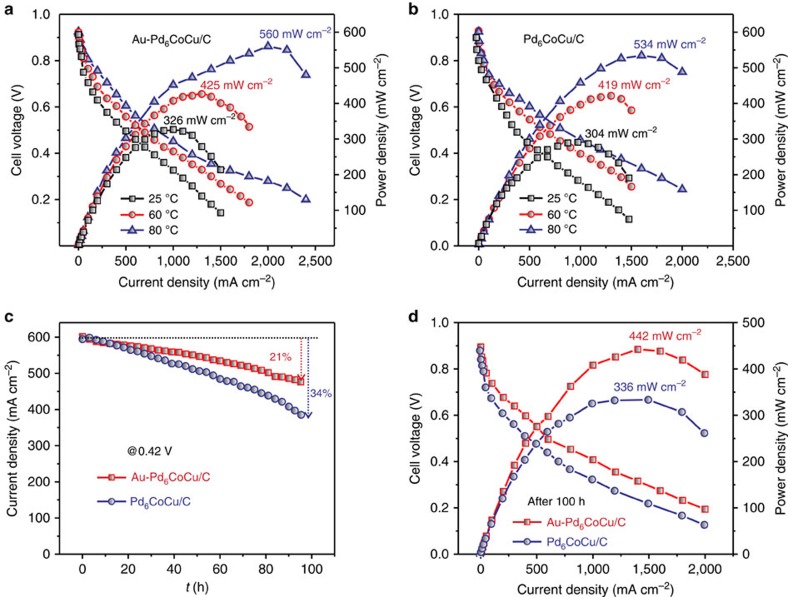
Single-cell performance. (**a**) Polarization and power-density curves for PEMFCs, using a Au–Pd_6_CoCu/C cathode catalyst at 25, 60 and 80 °C. (**b**) Polarization and power-density curves for PEMFCs using a Pd_6_CoCu/C cathode catalyst at 25, 60 and 80 °C. (**c**) Stability evaluation of Au–Pd_6_CoCu/C and Pd_6_CoCu/C cathodes on polarizing the cell at 0.42 V for 100 h in a single cell at 80 °C. (**d**) Comparison of the polarization and power densities for PEMFCs using a Au–Pd_6_CoCu/C and Pd_6_CoCu/C cathode catalyst after life test at 80 °C. Pt/C (Alfa Aesar, 40wt%) was used as the anode catalyst, and the metal loading was 0.5 mg cm^−2^ in both the anode and cathode. The current density values given are with respect to geometric electrode area.

The stability of the Au–Pd_6_CoCu/C cathode was further evaluated and compared with Pd_6_CoCu/C cathode in a single-cell PEMFC with commercial Pt/C as the anode (Fig. 7). Figure 7a shows the fuel-cell performance using Au–Pd_6_CoCu/C as the cathode electrocatalyst at different temperatures. It was observed that the fuel-cell performance was enhanced with increasing operating temperature. The maximum power density at 25 °C was 326 mW cm^−2^. It increased to 425 and 560 mW cm^−2^ at 60 °C and 80 °C, which is almost 30% and 72% higher than the starting value, respectively. This behaviour suggests that the oxygen reduction kinetics at the Au–Pd_6_CoCu/C nanoparticles were enhanced by increasing the temperature. The fuel-cell performance using Pd_6_CoCu/C as the cathode electrocatalyst at different temperatures exhibited similar behaviour as Au–Pd_6_CoCu/C (Fig. 7b). The long-term durability of the catalysts was assessed by recording the current density with time at a constant cell voltage of 0.42 V at 80 °C for 100 h (Fig. 7c) and the polarization and power-density curves before and after polarizing the fuel cell (Fig. 7d). The Au–Pd_6_CoCu/C cathode exhibited higher durability. The losses in the current density and in maximum power density were both about 21%. Compared with the losses of 34 and 37% in the current density and the maximum power density observed from the Pd_6_CoCu/C cathode, it further demonstrates that Au decoration enhanced the durability of Pd_6_CoCu/C cathode electrocatalysts.

## Discussion

The use of gold to stabilize fuel-cell nanocatalyts for the ORR has become an increasingly attractive strategy. However, developing a synthetic procedure that can lower Pt and Au usage, while preserving scalability for industrial production, has remained challenging and elusive. The method that we have developed, and which we present here, is extremely effective and scalable and addresses the above-mentioned challenges. Compared with methods pioneered by the Adzic group, which are only applicable to laboratory scale experiments, our strategy eliminates the need to carry out lengthy synthesis steps^36^ and Cu under-potential deposition^34^. In addition, we have demonstrated that only trace amounts of gold (Au:Pd=1:100) are needed to stablize the Pd-based alloy particles. This is almost a two-orders of magnitude lower loading of gold, compared with results reported in previous Au–Pd studies^29^,^36^. Moreover, according to the previous study^34^, we expected that the Pd_6_CoCu particles would have been decorated with a Au overlayer or Au clusters. Contrary to that expectation, in the present work we found that gold atoms actually penetrate the Pd–Co–Cu lattice and uniformly distribute within the particles without disrupting the host particle structure. Therefore, our strategy offers not only technological advances, but also undiscovered new structures and synthesis routes.

In summary, a ternary Pd_6_CoCu nanoparticle catalyst with ultralow amounts of Au decoration (Au–Pd_6_CoCu/C) has been successfully prepared by a simple spontaneous replacement method. Contrary to all previous examples, the Au is homogeneously distributed throughout the nanoparticles (over 10,000 cycles). The Au–Pd_6_CoCu/C catalysts exhibited excellent stability for the ORR under-potential cycling both in N_2_- and O_2_-saturated 0.1 M HClO_4_ solution. The single fuel-cell test indicates that the durability of Au–Pd_6_CoCu/C significantly enhances the 100-h life test compared with Pd_6_CoCu/C. The high durability of the electrocatalyst for the ORR can be ascribed to the homogeneous distribution of gold and the charge transfer of Pd to Au, causing a reduction of the *d*-electron occupation of Pd with a slightly expanded lattice, which enhances the corrosion resistance of Pd. This study provides a new strategy for optimizing the stability of fuel-cell catalysts.

## Methods

### Material synthesis

Carbon supported Pd_3_Co, Pd_3_Cu and Pd_6_CoCu nanoparticles with 20wt% of Pd were prepared using an impregnation method. In a typical synthesis for Pd_6_CoCu/C, 67 mg of PdCl_2_, 14.9 mg of CoCl_2_·6H_2_O and 10.7 mg of CuCl_2_·2H_2_O were first dissolved in deionized water, and 152 mg of preheated Vulcan XC-72 carbon support were dispersed in the solution. After ultrasonic blending for 30 min, the suspension was heated under magnetic stirring to allow the solvent to evaporate and to form a smooth, thick slurry. The slurry was dried in an oven at 60 °C overnight and grounded in an agate mortar, the resulting dark and free-flowing powder was then reduced in a tube furnace at 150 °C under flowing H_2_/N_2_ for 2 h. Pd_3_Cu/C and Pd_3_Co/C were prepared in the same procedure for comparison. The as-prepared Pd_3_Cu/C, Pd_3_Co/C and Pd_6_CoCu/C nanoparticles were heat-treated at 500 °C under a flowing H_2_ atmosphere for 10 h to form a Pd-rich shell.

The Au-decorated Pd_6_CoCu/C nanoparticles were prepared by a spontaneous displacement reaction. Fifty milligrams of the as-prepared carbon-supported Pd_6_CoCu/C nanoparticles were suspended in 10 ml of 0.1 mM NaAuCl_4_ solution. One monolayer of Au (calculated from stoichiometric ratios) was deposited on the Pd_6_CoCu/C nanoparticles surface (the atomic ratio of Pd to Au is ∼100:1). After ultrasonic blending for 30 min, the suspension was heated to 60 °C under magnetic stirring and left to react for 5 h. The sample was then centrifuged and washed using deionized water until the pH value was close to 7, and finally dried in a vacuum oven overnight.

### Characterization

The catalysts were characterized by using an X'Pert PRO diffractometer, and diffraction patterns were collected at a scanning rate of 4° per min. The STEM–EELS maps were acquired on a 5th order aberration-corrected STEM (Nion UltraSTEM) operated at 100 kV, with a convergence angle *α*_max_=∼30 mrad. The STEM–EDX line profile was acquired on an aberration-corrected JEOL ARM200CF operated at 200 keV with a Large Angle SDD-EDX detector, DrySD100GV. The effective detection area is 100 mm^2^. A nickel TEM grid was used to avoid background signals from Cu. The 2D STEM–EDX map was acquired on an aberration-corrected dedicated STEM (Hitachi HD2700C) operated at 200 keV with a Bruker SSD EDX detector. XPS data were obtained using an AXIS-ULTRA DLD-600 W Instrument.

### Electrochemical testing

Electrochemical experiments were carried out in 0.1 M HClO_4_ at room temperature using an Autolab electrochemistry station. Working electrodes were prepared by applying a thin catalyst film onto a glassy carbon electrode (GC, 5 mm in diameter). The catalyst ink was prepared by mixing 5 mg of the catalyst with 1 ml of Nafion (0.05wt% Nafion dissolved in ethanol) solution. The mixture was sonicated and about 5.0 μl were applied onto a glassy carbon disk. After solvent evaporation, a thin layer of the Nafion-catalyst-Vulcan ink remained on the GC surface to serve as the working electrode. The Pd loading on the rotating disk electrode was calculated as 25.5 μg_Pd_ per cm^2^. A Pt wire was used as the counter electrode and a reversible hydrogen electrode, in the same electrolyte as the electrochemical cell, was used as the reference electrode. All potentials are referred to the reversible hydrogen electrode. The ORR polarization curves were obtained by sweeping the potential from +0.20 to +1.0 V at a scan rate of 5 mV s^−1^ and at a rotation rate of 1,600 r.p.m.

### MEA preparation and electrochemical investigation in single cells

Membrane electrode assemblies (MEAs), with an active electrode area of 1 cm^2^, were prepared using the gas-diffusion electrode method. Pt/C (Alfa Aesar, 40 wt%) was used as the anode and the as-prepared Pd_6_CoCu/C and Au–Pd_6_CoCu/C nanoparticles were used as the cathode catalysts. The catalyst loading was 0.2 mg_Pt_ per cm^2^ on the anode and 0.25 mg_Pd_ per cm^2^ on the cathode. The anode and cathode gas-diffusion electrodes were placed on the two sides of a Nafion 211 membrane (DuPont), and then were hot-pressed with a pressure of 0.5 MPa for 1 min at 140 °C to form an MEA. The MEA was inserted into a single-cell module for testing. The single cell was activated at a cell temperature of 80 °C. Fully humidified H_2_ and O_2_ were supplied to the anode and the cathode with a flow rate of 300 and 500 s.c.c.m., respectively. During the activation process, the current density was held constant for 30 min every time it reached 0.5, 1, 1.5 and 2 A cm^−2^. Polarization curves were recorded in a galvanostatic mode (without ohmic; iR-correction) with a hold time of 3 min per point. To study the temperature effects on the cell performance, polarization curves were recorded at 25, 40, 60 and 80 °C. Durability tests were performed at a constant potential of 0.42 V for 100 h of polarization after conditioning as described above without interruption. The humidity and temperature were kept the same as during the polarization tests.

### Data availability

The authors declare that all the data supporting the findings of this study are available within the article and its Supplementary Information files.

## Additional information

**How to cite this article:** Wang, D. *et al*. Spontaneous incorporation of gold in palladium-based ternary nanoparticles makes durable electrocatalysts for oxygen reduction reaction. *Nat. Commun.* 7:11941 doi: 10.1038/ncomms11941 (2016).

## Supplementary Material

Supplementary Information Supplementary Figures 1-6 and Supplementary Table 1
