# Supplementary material for: Spontaneous incorporation of gold in palladium-based ternary nanoparticles makes durable electrocatalysts for oxygen reduction reaction
Source: Nat Commun. 2016 Jun 23;7:11941. doi: 10.1038/ncomms11941 (PMC4931015; doi:10.1038/ncomms11941)
Supplement: Supplementary Information — Supplementary Figures 1-6 and Supplementary Table 1 [file ncomms11941-s1.pdf]

Supplementary Information

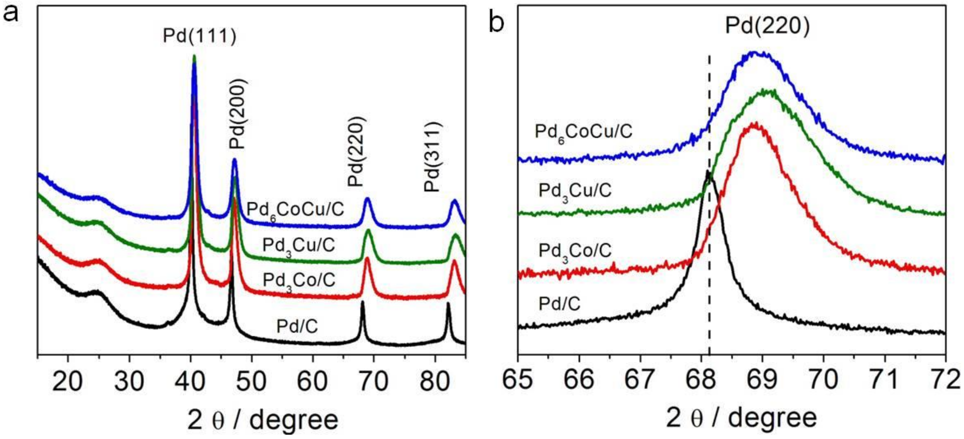

Supplementary Figure 1. (a) XRD patterns for Pd/C, Pd_3Co/C, Pd_3Cu/C and Pd_6CoCu/C, (b) Expanded (220) diffraction peaks.

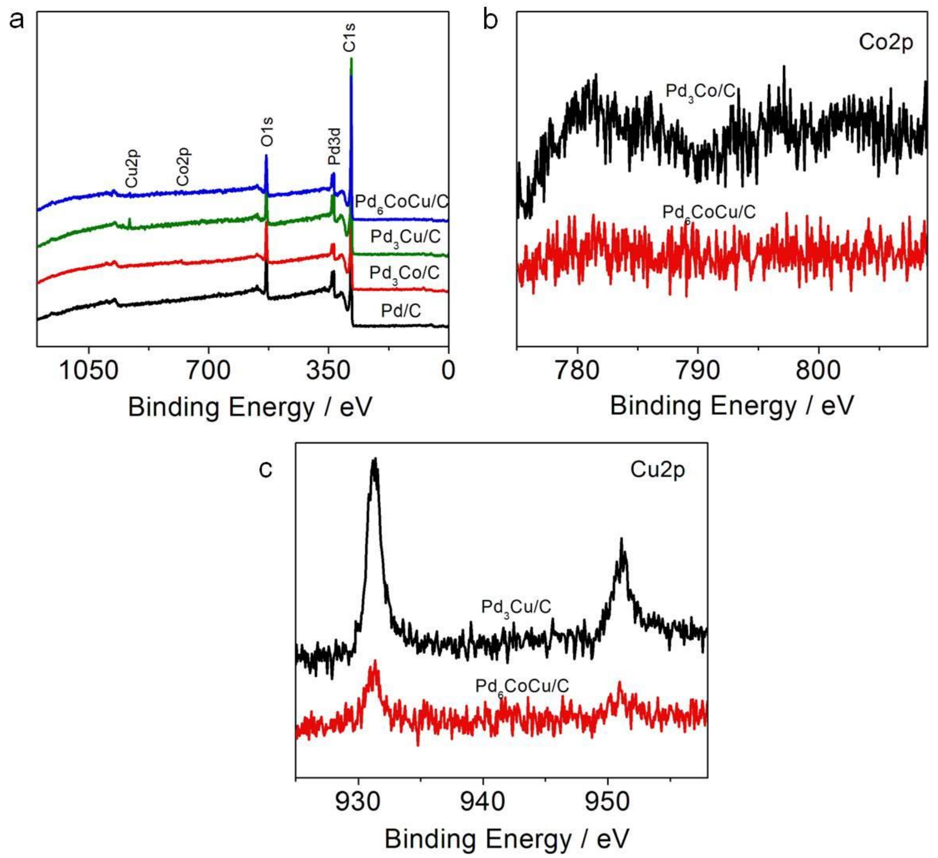

Supplementary Figure 2. (a) Survey spectrum XPS of Pd/C, Pd_3Co/C, Pd_3Cu/C and Pd_6CoCu/C. (b) Fine spectrum of Co2p. (c) Fine spectrum of Cu2p.

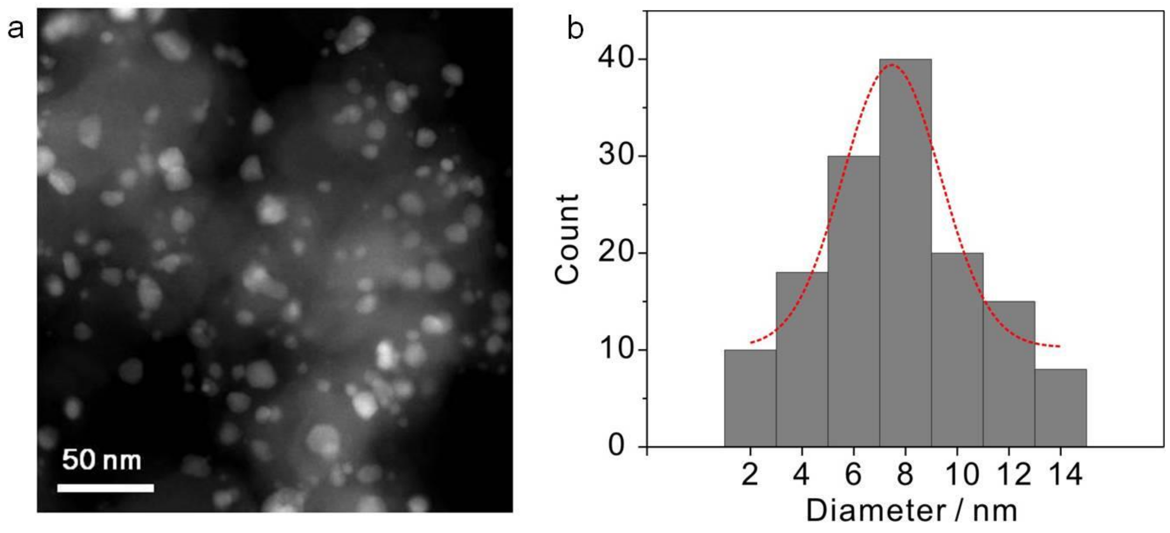

Supplementary Figure 3. (a) Overview ADF-TEM image of Pd_6CoCu/C. (b) The particle size distribution.

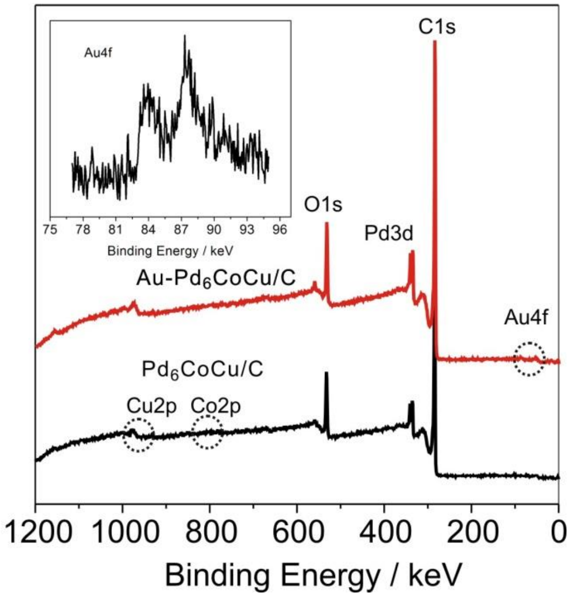

Supplementary Figure 4. Survey spectrum XPS of Pd_6CoCu/C and Au-Pd_6CoCu/C. The inset shows the fine spectrum of Au4f

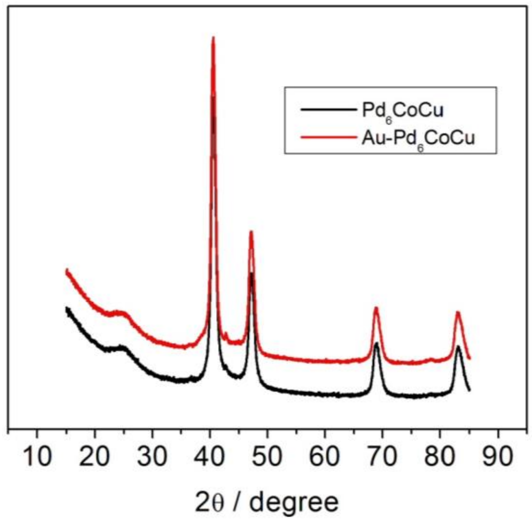

Supplementary Figure 5. XRD patterns for Pd_6CoCu/C and Au-Pd_6CoCu/C.

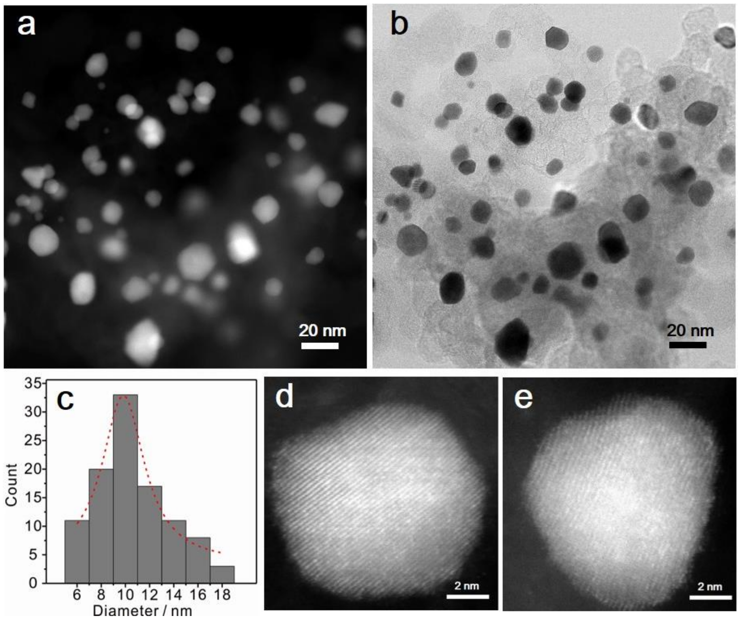

Supplementary Figure 6. (a) Overview DF-TEM image and (b) BF-TEM image of Au-Pd_6CoCu/C nanoparticles after 10,000 potential cycles. (c) The particle size distribution. (d,e) Two HRTEM images of the particles.

Supplementary Table 1. XRD Results of Pd-based nanoparticles

Sample	2θ (220) deg	domain size nm	lattice parameter nm
Pd/C	68.168	24	0.3887
Pd_3Co/C	68.939	15	0.3848
Pd_3Cu/C	69.126	12	0.3839
Pd_6CoCu/C	68.996	8	0.3846
Au-Pd_6CoCu/C	68.927	7.8	0.3849
